# Introduction of Cellulolytic Bacterium *Bacillus velezensis* Z2.6 and Its Cellulase Production Optimization

**DOI:** 10.3390/microorganisms12050979

**Published:** 2024-05-13

**Authors:** Zhi Cai, Yi Wang, Yang You, Nan Yang, Shanshan Lu, Jianheng Xue, Xiang Xing, Sha Sha, Lihua Zhao

**Affiliations:** 1 SDU-ANU Joint Science College, Shandong University, Weihai 264209, China; zchua_02@mail.sdu.edu.cn (Z.C.); 202000700288@mail.sdu.edu.cn (Y.W.); 202100700291@mail.sdu.edu.cn (Y.Y.); 202000700294@mail.sdu.edu.cn (N.Y.); 202100700258@mail.sdu.edu.cn (S.L.); xjh-in-sdu@mail.sdu.edu.cn (J.X.); xingxiang@sdu.edu.cn (X.X.); 2 Marine College, Shandong University, Weihai 264209, China

**Keywords:** isolation, *Bacillus velezensis*, cellulolytic bacteria, genome analysis, cellulase, optimization

## Abstract

Enzyme-production microorganisms typically occupy a dominant position in composting, where cellulolytic microorganisms actively engage in the breakdown of lignocellulose. Exploring strains with high yields of cellulose-degrading enzymes holds substantial significance for the industrial production of related enzymes and the advancement of clean bioenergy. This study was inclined to screen cellulolytic bacteria, conduct genome analysis, mine cellulase-related genes, and optimize cellulase production. The potential carboxymethylcellulose-hydrolyzing bacterial strain Z2.6 was isolated from the maturation phase of pig manure-based compost with algae residuals as the feedstock and identified as *Bacillus velezensis*. In the draft genome of strain Z2.6, 31 related cellulolytic genes were annotated by the CAZy database, and further validation by cloning documented the existence of an endo-1,4-β-D-glucanase (EC 3.2.1.4) belonging to the GH5 family and a β-glucosidase (EC 3.2.1.21) belonging to the GH1 family, which are predominant types of cellulases. Through the exploration of ten factors in fermentation medium with Plackett–Burman and Box–Behnken design methodologies, maximum cellulase activity was predicted to reach 2.98 U/mL theoretically. The optimal conditions achieving this response were determined as 1.09% CMC-Na, 2.30% salinity, and 1.23% tryptone. Validation under these specified conditions yielded a cellulose activity of 3.02 U/mL, demonstrating a 3.43-fold degree of optimization. In conclusion, this comprehensive study underscored the significant capabilities of strain Z2.6 in lignocellulolytic saccharification and its potentialities for future in-depth exploration in biomass conversion.

## 1. Introduction

Biofuel represents a form of sustainable and environmentally benign energy that is the subject of ongoing and dedicated pursuit within the scientific community. The growing shortage of fossil fuels requires the search for and utilization of such renewable bioenergy from fermentable polysaccharides in which lignocellulose, majorly composed of cellulose, is a considerable source to realize this substitution [1,2]. As the most abundant organic matter on earth, cellulose is a linear macromolecular polysaccharide composed of D-glucose with β-1,4-glycosidic linkages, encompassing the cell walls of both wood and plants, as well as microbial-based cellulose [1]. Embraced in the saccharification and fermentation process, cellulose degradation is of great importance in the treatment of lignocellulosic biomass, such as agricultural byproducts and wastes [3,4]. This bioconversion exhibits advantages in preserving the original carbohydrate structures and simultaneously reacting with efficiency in moderate conditions. However, the degradation and conversion of these cellulose-abundant biomasses are inadequate and inefficient, highlighting an urgent need for more effective cellulose decomposition strategies [5,6]. Microbial cellulases act as crucial candidates to resolve this matter, which may revolutionize the production of sustainable biofuels, streamline industrial processes such as textile and paper manufacturing, and contribute to environmentally friendly solutions for biomass utilization [2,4].

Compost potentially serves as a valuable reservoir for cellulolytic enzymes, which are produced by a diverse array of cellulose-degrading microorganisms. As one particular bioconversion process involving massive lignocellulosic ingredients, composting is a desirable approach to realize the recycling of organic wastes, and microbes play an essential role during the process, possessing high biological diversity and activities [7]. Among various substrates, algae, even if important in stabilizing the marine environment, can also result in residual biomass; nevertheless, the feasibility of composting algae wastes has been substantiated and promises uses in fertilizer, biorefinery, and microbial applications [8]. Currently, novel *Bacillus* strains and functional microbes, isolated from municipal waste compost for their polysaccharide degrading, may significantly contribute to future research in bioagent development and enzyme production [7,9,10].

In the context of the microbial utilization of cellulose, the saccharification of cellulosic materials is facilitated by the enzymatic functions of three primary cellulase types, namely endo-1,4-β-D-glucanase (endoglucanase, EC 3.2.1.4), exo-1,4-β-D-glucanase (exoglucanase, EC 3.2.1.91), and β-glucosidase (EC 3.2.1.21) [2,6]. Corresponding organisms capable of cellulolysis have been identified as a source of diverse cellulases and their novel derivatives, whose depolymerization efficiency or stress resistance is also in sight [6,11]. The utilization of these cellulose-decomposing enzymes presents promising prospects in the biorefinery and biologics industries [12]. Among the numerous beneficial attributes of such organisms, *B. velezensis* displays a wide spectrum of abilities in biocontrol, hydrolase production, and probiotics, all of which have substantial applications in the agriculture industry and relative utilization in pharmaceutics [13,14,15,16]. Nonetheless, recent applications of *B. velezensis* from recycled compost for secondary usage following fermentation have not been explored much. Potential uses for cellulase applications have been elucidated in different strains of *B. velezensis*, and comparative genomics highlights similarities among these different strains [17,18,19]. However, due to the contingency and incompleteness of current knowledge, further inquiries and research are still needed to validate the anticipated gene expression, thereby contributing to industrial lignocellulose treatment.

In this article, we isolated a cellulose-degrading strain, designated as Z2.6, from the maturation stage of compost derived from algae residuals and pig manure, with thorough identification conducted on taxonomy. The genomic studies predicted coding sequences for cellulolytic enzymes, revealing the strain`s cellulolytic potential. To further enhance cellulase production, the response surface method (RSM) was meticulously employed for the medium environment in which a combination of processive statistical analyses, including single-factor experiments, Plackett-Burman (PB) experimentation, path of steepest ascent/descent design, and Box-Behnken design (BBD), have been conducted. This comprehensive trial identified specific microorganisms capable of cellulose degradation in the recycled compost and laid the foundation for future exploitation of such strains, opening avenues for efficient and convenient applications, such as enzyme production, compost inoculants, and bioagents.

## 2. Materials and Methods

### 2.1. Microorganism, Compost Sample, and Isolation

Bacterium *B. velezensis* Z2.6 was isolated from a compost sample in our laboratory and used in this study. The original compost of manure-based and algae residual mixtures was accomplished at Weihai, Shandong, China (37°30′7″ N 122°7′24″ E). After warming for approximately 8 days in the maturation phase, the compost sample was stored at −80 °C before screening for cellulose-degrading bacteria. During the enrichment stage, 1.0 g of fresh compost sample was collected in a 5 mL aseptic screw tube. This sample was then suspended in 150 mL sterile conical flask containing 50 mL enhancement medium at 150 rpm and 40 °C for 48 h as bacterial suspension. Media with sodium carboxymethyl cellulose (CMC-Na) were modified according to Hungate [20], and here this enhancement broth was made up of CMC-Na 10 g, KH_2_PO_4_ 1.31 g, KNO_3_ 3.0 g, NaCl 0.5 g, MgSO_4_·7H_2_O 0.5 g, and FeCl_3_ 0.05 g, which were dissolved in a 1.0 L of mixture of 1:1 (*v*/*v*) distilled water and aged seawater from coastal Weihai.

Next, an enriched bacterial suspension from the above media was homogenized and sterilely diluted up to 10^−5^, 10^−6^, and 10^−7^, and 50 μL of each diluent was spread on CMC-Na agar (CA), which contained 10 g CMC-Na, 1.31 g KH_2_PO_4_, 3.0 g KNO_3_, 0.5 g KCl, 0.5 g MgSO_4_·7H_2_O, 0.05 g FePO_4_, and 18 g agar in 1.0 L of the same mixture above at pH 6.8. All media in this study were sterilized at 121 °C for 15 min before storage or use. Culturing at 40 °C for 3 days with morphological observation and pigmentation, each representative type of colony was continuously streaked on fresh CA for pure culture.

### 2.2. Screening of Cellulolytic Bacteria and Maintenance Conditions

Isolated colonies were roughly qualitatively screened for cellulose-degrading ability with the protocol of Teather and Wood [21]. Single, well-growing, individual colonies in each pure culture were further inoculated onto CA, where three purified same colonies were point-planted on CA with the remaining quarter area remaining as blank control. After culturing at 40 °C for 24 h, 48 h, 72 h, and 96 h, these plates were flooded with 0.1% Congo red solution for 15 min followed by washing with 1 M NaCl for 10 min. The hydrolyzed zone (H) produced around each colony and its size (C) were measured [22] and were converted into the H/C ratio as the average in triplicates. Those strains with better H/C values and growing status were selected for secondary selection by assessing cellulase-degrading ability after CMC-Na broth (CB) cultivation, where no agar was added in CA. For the final decision for optimization, strain Z2.6 of the maximum activity was chosen. All selected strains were stored at −80 °C in sterile 15% (*v*/*v*) glycerol supplemented with 1% NaCl.

### 2.3. Morphological, Physiological, and Biochemical Analysis and Growth Curve

The morphological, physiological, and biochemical parameters of strain Z2.6 were examined after 48 h cultivation at 40 °C on Luria–Bertani (LB) agar (for 1 L medium: yeast extract 5.0 g, tryptone 10.0 g, NaCl 10.0 g; pH 7.0). By consulting the Common Bacterial System Identification Manual [23], the morphological features were included (size, shape, color, margin, texture, and viscosity) and then Gram staining was also performed for identification. To identify physiological and biochemical traits of the strain Z2.6, its pure suspension, prepared from LB agar, was inoculated into an ampoule bottle with indicator solution (from Hopebio Co., Ltd., Qingdao, China) or tested by standard methods [23] for scheduled test time. This identification process encompassed a series of tests, incorporating assessments for catalase, Voges–Proskauer (VP) test, and methyl red test, as well as tests to ascertain the utilization of sugar alcohols (such as lactose, glucose, mannose, sorbitol, salicin), amino acids (lysine, arginine, ornithine, and indole), and acid salts (propionate, malonate, citrate). Furthermore, investigation into the heat tolerance, resistance to ambient pH, and salt (NaCl) resistance were conducted to assess Z2.6’s growth conditions.

To characterize the growth features of Z2.6, a single colony was selected to inoculate into LB broth to prepare the seed culture solution. Subculturing was performed weekly by transferring 1 mL of seed solution to 100 mL fresh medium at 40 °C with shaking. Fresh media were inoculated with 2 mL seeds, and the optical density (OD) at 600 nm was measured by intervals of 2 h, further increasing to 4 h [24]. Furthermore, the growth curve was plotted in R 4.2.0 with average OD_600 nm_ as the representative for the growth amount.

### 2.4. 16S rRNA Gene Sequencing and Phylogenetic Analysis

The 16S rRNA gene fragment was amplified with primers 27F (5′-AGAGTTTGATCCTGGCTCAG-3′) and 1492R (5′-TACGGCTACCTTGTTACGACTT-3′) from the genome of strain Z2.6 by spin column extraction. The PCR products were purified by agarose gel electrophoresis and sequenced by BGI Co., Ltd. (Wuhan, China). The returned 16S rRNA alignment was BLASTed against GenBank (https://blast.ncbi.nlm.nih.gov/Blast.cgi, accessed on 5 March 2023) and EzBiocloud (http://www.ezbiocloud.net, accessed on 5 March 2023) to calculate sequence similarity and identify closely related type strains [25]. Multiple sequence alignments and phylogenetic tree construction were implemented by ClusalW and maximum-likelihood estimation in MEGA version 11.0 [26], and the stability of the topology confirmation was performed with bootstrap analyses based on 1000 replications [27].

### 2.5. Enzymatic Assay

#### 2.5.1. Extraction of Crude Enzyme Solution

Initially, cellulolytic bacteria were activated and cultured in seed media CB. Later, a modification was made to transform it into a fermentation medium (FM) with basic composition as CMC-Na 10 g, tryptone 3.0 g, NaH_2_PO_4_ 1.0 g, KCl 0.5 g, MgSO_4_·7H_2_O 0.5 g, and FePO_4_·4H_2_O 0.05 g for continuous optimization. To control the inoculation concentration, strain Z2.6 was activated in LB broth and cultured in a constant temperature shaker at 150 rpm and 40 °C for 12 h. This stock solution was sterilely adjusted to OD_600 nm_ of 1.0 using fresh LB broth. Subsequently, 1.0 mL of the stock solution was inoculated into 50 mL fresh CB or other fermented media in a 150 mL conical flask and cultured for a predetermined period, typically 48 h or 72 h. Finally, the crude enzyme solution was obtained from the superstratum of the CB after centrifugation at 12,000× *g* rpm and 4 °C for 15 min. Temporary storage was at 4 °C before cellulase activity assay.

#### 2.5.2. Crude Cellulase Activity Assay

Cellulase activity was measured with the 3,5-dinitrosalicylic acid (DNS) colorimetric method, which was adapted from the approach outlined by Ghose [28] and Miller [29]. The absorbance post-reaction was measured at 540 nm, OD_540 nm_, against a blank control. Enzyme activities were subsequently determined by referencing a standard curve constructed with different concentrations (*w*/*v*) of glucose. Determination of the optimum enzyme reaction condition was estimated in prior assays with a gradient as 20–70 °C and pH 3.0–10. Cellulase activity was defined in international units (U), representing the amount of enzyme required for reducing CMC-Na to produce one μmol of glucose per minute [28]. The results were expressed in mean ± SEM and analyzed in R.

### 2.6. Genome Sequencing, Annotation, and Functional Analysis

The strain Z2.6 was cultured in LB broth for 48 h and then the genomic DNA was extracted by using a Bacteria DNA kit ( Takara, Kusatsu, Japan) according to the manufacturer’s instructions. The harvested DNA was detected by agarose gel electrophoresis and quantified by Qubit^®^ 2.0 Fluorometer (Thermo Scientific, Waltham, MA, USA) and was further fragmented to a size of 350 bp with modification for generating sequencing libraries by NEBNext^®^ Ultra™ DNA Library Prep Kit for Illumina (NEB, Ipswich, MA, USA). The whole genome of Z2.6 was further sequenced by Beijing Novogene Bioinformatics Technology Co., Ltd. (Beijing, China), using the Illumina Hiseq Xten platform (Illumina Inc., San Diego, CA, USA). The raw sequencing data were filtered by step for quality control by using Illumina base-calling software CASAVA v1.8.2 (http://www.support.illumina.com, accessed on 5 May 2023). All good-quality paired reads were assembled using the SOAP denovo software (version 2.04) into a number of scaffolds [30]. The genome component prediction was performed with NCBI prokaryotic genome annotation pipeline (PGAP) [31], GeneMarkS program (version 4.17) [32], and RNAmmer software (version 1.2) [33] to predicted coding genes and ncRNA. The 16S rRNA was compared between the PCR amplification and that obtained from the genome by RNAmmer 1.2 server for authenticity. The function prediction was conducted by using Rapid Annotations using Subsystem Technology server (RAST) [34], Kyoto Encyclopedia of Genes and Genomes (KEGG) database [35], Prokka software [36], and Cluster of Orthologous Groups of proteins (COG) [37]. The Carbohydrate-Active enZYmes (CAZy) database [38] was used to predict the carbohydrate-related enzymes and their coding sequences. Further investigation of cellulase-related enzymes was predicted by dbCAN3 against HMMER, DIAMOND, and eCAMI, with default parameters [39]. These predicted CAZymes associated with cellulase in strain Z2.6 were identified and filtered using BLAST against Swiss-Prot (http://www.uniprot.org/blast, accessed on 23 March 2024). Extracting from the genome, 5 corresponding GH family sequences were cloned to validate their existence and accurate coding information (with primers in Table S1).

To further detect the taxonomic phylogeny of strain Z2.6, the genome sequence was uploaded to the Type (Strain) Genome Server (TYGS) to build a whole-based tree. Additional pairwise whole genome comparisons of average nucleotide identity (ANI) and average amino acid identity (AAI) were generated with tools from Rodriguez-R and Konstantinidis (http://enve-omics.ce.gatech.edu/ani/index, accessed on 2 March 2024). The calculated results were disposed in R to construct the ANI/AAI matrix.

### 2.7. Statistical Design for Process Optimization

#### 2.7.1. Complete Randomized Design

With the cellulase active strains screened by the indicators H/C ratio and cellulase activities, the heterotrophic strain Z2.6, due to its potentiality from compost, was chosen for continuous promotion of cellulase production based on FM. The procedure here is shown in Table S2 and was first aimed at determining the relative optimized carbon and nitrogen sources, with further conditions depicted under these two decided sources. Following assays for each factor among these 8 independent parameters, some were directly adjusted one individual factor at a time in gradients (Table S2), while others were kept intact. Moreover, the execution orders were ranked as CMC-Na concentration (*w*/*v*), tryptone concentration (*w*/*v*), initial pH, temperature, salinity indicated by NaCl (*w*/*v*), culturing time, inoculum size (*v*/*v*), and media bottling size. Of note, factors excluding time were measured at 2 points in time at 48 h and 72 h, and 150 mL flasks were used for liquid culture through this design. Finally, results were loaded to R for two-way ANOVA analysis.

#### 2.7.2. Plackett–Burman Design

The PB design serves as an effective technique for selecting significant parameters during the optimizing of cellulase production [40]. Following the principles of PB design, each parameter was evaluated at two levels: low and high, denoted by (−) and (+) signs, respectively. Moreover, these factors were narrowed down to the three most significant ones, which were further fitted into a linear regression. Table S3 lists the eight parameters involved, encompassing chemical factors (such as concentration of carbon or nitrogen source), physical factors (including temperature, pH, salinity, and incubation time), and biological factors (inoculum size and medium bottling size). The average of triplicates was taken for all the runs to represent the response.

#### 2.7.3. Path of Steepest Ascent

The significant factors at the 95% confidence level (*p* < 0.05) in the PB design were identified and subsequently optimized using the steepest ascent/descent method. Experiments were conducted along the steepest ascent/descent with defined intervals, which were estimated based on the coefficient ratio and by practical experience [41]. Three factors as CMC-Na, salinity, and tryptone were selected for the steepest ascent/descent experiment.

#### 2.7.4. Response Surface Construction

After determining the most significant variables and setting a central point with the above protocols, the Box–Behnken design was employed to evaluate the optimal levels and interactions of these three key variables [42]. The variables, along with their levels, are presented in Table S4. A total of 17 experimental runs were implemented to derive a second-order non-linear polynomial regression model. Design Expert (version 13.0, Stat-Ease Inc., Minneapolis, MN, USA) was used for PB design and BBD. The models were statistically analyzed by analysis of variance (ANOVA), *p*-values, and *F*-values. The optimum values of the three variables were calculated from three-dimensional (3D) response surface. Subsequently, tests were conducted to validate the predicted cellulase activities under permissive fermentation conditions using the statistical model.

## 3. Results and Discussion

### 3.1. Microorganism Screening and Strain Characteristics

In total, 56 strains of cellulolytic bacteria were isolated from the compost sample. The strain used for this study was designated as Z2.6 (=CGMCC 28578), which grows well on CA (Figure S1a). In morphology, its colonies were creamy white, rough, with minor translucence, and uplifted with clear edges. Under light microscopy by Gram staining (Figure S1c), Z2.6 was Gram-positive and short and rod-shaped with endospores forming. This strain was initially selected based on the comparison of the cellulose hydrolysis zone (H) to the colony size (C), with reference to the superiority in H/C ratio and increasing speed along time [14]. Strain Z2.6 met both parameters with final average H/C being 5.45 ± 0.82 on day 5 (Table S5). Consequently, this isolated strain with relatively high cellulase production was confirmed for further exploration.

Physiological and biochemical tests on Z2.6 (Table 1) clarified utilization of lactose, sucrose, glycerol, mannose, and esculin, with completing gelatin liquefaction. However, negative reactions were tests of sorbitol, salicin, dulcitol, raffinose, and propionate, indicating inabilities to produce corresponding enzymes. The results for malonate and Simon`s citrate salt demonstrated strain Z2.6 failed to grow with malonate or citrate as the sole carbon source. Identification tests, namely the VP test, indole, and methyl red, were all negative, with the remaining catalase performing as positive. Furthermore, strain Z2.6 was found incapable of producing urease, lysine decarboxylase, ornithine decarboxylase, and arginine double hydrolase while exhibiting activity on β-galactosidase due to the positive reaction to ONPG (Table 1). When inoculated in CB, this strain could tolerate temperature to approximately 65 °C, and its minimum growth temperature was 10 °C (Table 1). Additionally, its salt tolerance may be higher than 8.0% NaCl, close to its heterotypic synonym *B. methylotrophicus* RYC01101 [43]. According to the Bergey’s Manual of Determinative Bacteriology [44] and the Common Bacterial System Identification Manual [23], the above results for physiological and biochemical characteristics exhibited a great similarity to the Gram-positive bacterium *Bacillus* sp. To gain a schematic view on Z2.6 growth, a growth curve was drawn and matched to a logistics curve through 15 time intervals by shaking flasks and continuous density measuring (Figure S1g). The growth curve was well fitted to this model, with three parameters being significant and achieving convergence tolerance ($3.11\times 10^{-6}$, Table S6) and also providing a nonsignificant difference between the observed and predicted measurements (*p* > 0.23). In the growth curve, there was a delayed growth period from approximately 0 h to 3 h, followed by a logarithmic growth phase lasting 9 h. This elucidated that incubation time of 10 h could be chosen for the seed culture. With integrated similarities to *B. velezensis* strain M2 in growth status [5], both curves in LB shared a plateau period from 20 h to 40 h. However, this curve was measured in flasks with a larger volume of media, providing a more suitable reference for relatively extended cultivation.

### 3.2. Identification and Phylogenetic Analysis of Strain Z2.6

**Figure 1:**
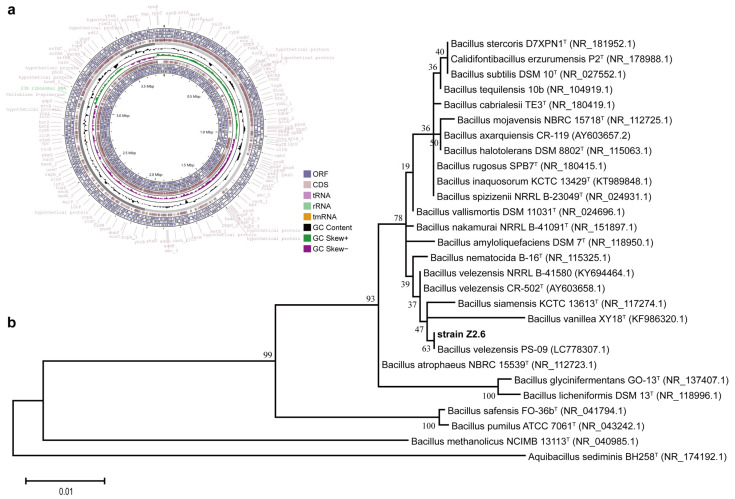
Genome map and phylogenic tree of strain Z2.6. (**a**) Circular genome map of strain Z2.6. The circular illustration containing five groups of rings was constructed by CGView Sever. Six open reading frame (ORF) rings (in lilac) and two coding sequence (CDS) rings (in light pink) were categorized as two sets in forward and reverse strand directions, respectively located at the outermost and the innermost circular areas. The rings between are aligned as scaffolds, GC content, and GC skew from the outside to the inside. Moreover, the tRNA, rRNA, and tmRNA genes are respectively presented by pink, light green, and orange arrows. (**b**) Maximum-likelihood phylogenetic tree based on 16S rRNA gene sequences of strain Z2.6 and representative of the other members of close-affinity species in genera bacillus. Bootstrap values (>40) are shown at branch nodes (ML/NJ/MP) based on 1000 replicas. *Aquibacillus sediminis* BH258 (GenBank accession number NR_174192.1) was chosen as an outgroup. Bar = 1% estimated sequence divergence as substitutions per nucleotide position.

The 16S rRNA gene BLAST results showed that the strain Z2.6 exhibited the highest sequence similarity to *B. velezensis* CR-502^T^ (AY603658) and *B. siamensis* KCTC 13613^T^ (NR_117274.1) with 99.85% similarity, followed by *B. subtilis* NCIB 3610^T^ (99.71%, ABQL01000001) and *B. amyloliquefaciens* DSM7^T^ (99.63%, NR_151897.1). Meanwhile, the maximum-likelihood phylogenetic tree of Z2.6 is shown in Figure 1b, indicating *B. velezensis* PS-09 (LC778307) has the closest phylogeny to strain Z2.6, with other strains CR-502^T^ and NRRL B-41580 in similar relationships.

**Figure 2:**
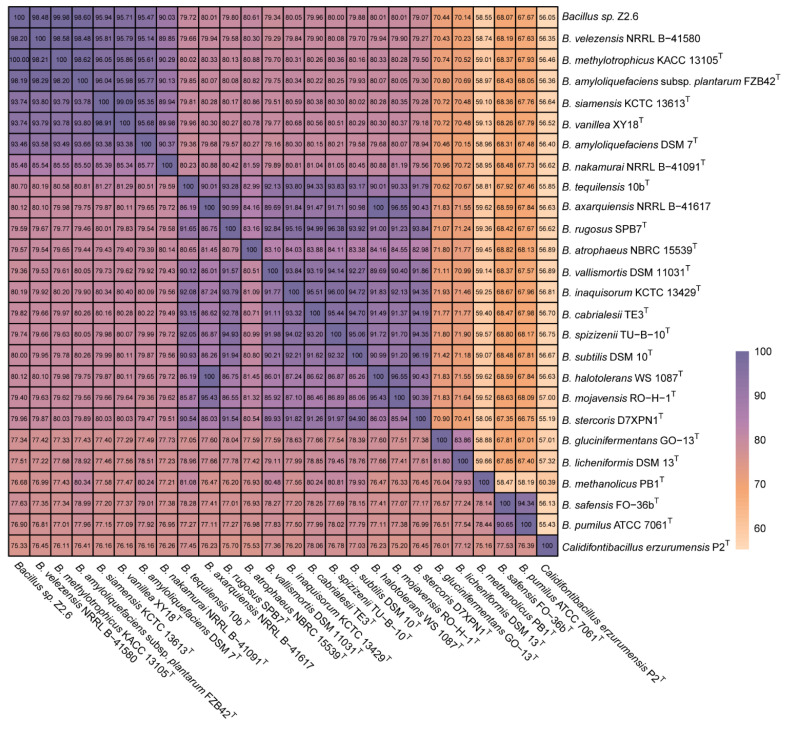
The nucleotide identity (ANI) and the average amino acid identity (AAI) combined heatmap. Pairwise comparisons of ANI and AAI between strain Z2.6 and reference genomes in genus *Bacillus* and a close strain *Calidifontibacillus erzurumensis* P2^T^. Upper triangle, AAI outcomes; lower triangle, ANI outcomes.

However, a Genome BLAST Distance phylogeny (GBDP) tree (Figure S2) implicated closer identities to *B. methylotrophicus* KACC 13105^T^, in which the contradiction was probably attributed to the complicated taxonomy of *B. velezensis* against *B. methylotrophicus* and *B. amyloliquefaciens*. Of note, the previous characterized novel strains, *B. methylotrophicus* KACC 13105^T^ and *B. amyloliquefaciens* subsp. *plantarum* FZB42^T^, were both heterotypic synonyms to *B. velezensis* CBMB205 (=KACC 13105^T^) and FZB42^T^ [16,45]. A further efficient verification was provided by ANI/AAI matrix (Figure 2), and the smallest ANI/AAI scores were calculated as 98.19 and 98.48, respectively. Hence, evidence from both 16S rRNA and comparative genomic assays finally suggested that strain Z2.6 represents a *B. velezensis* strain. According to previous studies, *B. velezensis* was initially isolated and characterized by Ruiz-García et al. [46] and named CR-502^T^ and CR14b in 2005. Succedent sources in aqueous systems included marine aquaculture [47] and fish intestines [48]. Other terrestrial sources were roots [49], soil [48,50], and waste [49]. Notably, Li et al. [5] and Khalid et al. [19] have successfully isolated strain M2 and Y1, respectively, from the manure of piglets. However, in our study, the origin was the maturation stage of compost samples containing algae residuals and pig manure, which correspondingly represented both marine and terrestrial sources. The wide distribution of *B. velezensis* may facilitate its bio-functional roles in various circumstances.

### 3.3. Genome Features and Mining of Potential Cellulases

The genome of strain Z2.6 has been assembled into five contigs and five scaffolds with a total length of 3,795,488 bps, a scaffold N50 value of 2,036,077 bps, and a scaffold L90 value of 418,559 bps (Figure 1a). The coding regions made up to 90.59% of the genome with 46.44% G+C content. There were 3919 genes with 877 bps in average length, and 62 ncRNA were predicted in the genome of strain Z2.6. Repetitive sequences as scattered ones and tandem ones in the Z2.6 genome accounted for 0.3032% and 0.2798%, respectively. In total, 10 genomic islands were annotated, of which 7 of them were longer than 15 kb. These repetitive sequences and genomic islands in bacterium are of great importance in the regulation of gene expression and acquiring of versatile characteristics [51]. This whole genome has been deposited in the DDBJ/ENA/GenBank under the accession number JBANFQ000000000.

The predictions of genomic functions showed 292 genes associated with amino acid transport and metabolism, 253 genes related to carbohydrate transport and metabolism, and 179 genes correlated with coenzyme transport and metabolism based on the COG database (Figure S3). The KEGG annotations exhibited that the strain Z2.6 had complete enzyme chains for the TCA cycle, pentose phosphate cycle, Embden–Meyerhof pathway, and gluconeogenesis pathway. Predictions were also made for D-galacturonate degradation, galactose degradation, and formaldehyde assimilation capabilities. The strain Z2.6 was predicted to conduct assimilatory nitrate reduction with *NasAB* (EC 1.7.99) and *NasBDE* (EC 1.7.1.4) to convert nitrate into ammonia. The strain Z2.6 was also predicted to reduce assimilatory sulfate with *Sat* (EC 2.7.7.4), *CysC* (EC 2.7.1.25), *CysH* (EC 1.8.4.8), and *CysJI* (EC 1.8.1.2) genes to covert sulfate into sulfide. The secondary metabolite predictions showed that the strain Z2.6 could produce bacilysin, lanthipeptide, betalactone and bacteriocin (Figure S4). Based on the CAZy database (Figure S5), the strain Z2.6 had 12 carbohydrate esterase genes (CEs), 69 glycoside hydrolase genes (GHs), 34 glycosyl transferase genes (GTs), and three polysaccharide lyase genes (PLs).

Further detection of potential genes involved in lignocellulose saccharification was conducted in accordance with the CAZy database, resulting in the enumeration of 31 annotated proteins (Table S7). This list compromised two endoglucanases (EC 3.2.1.4) from the GH51 family and nine β-glucosidases (EC 3.2.1.21), among which seven and two were categorized in families GH1 and GH3, respectively. The co-existence and approximate quantities of these annotated related proteins indicated the potential of strain Z2.6 in cellulose degradation, consistent with other *B. velezensis* strains such as FZB42^T^ and SSF6 [14]. To validate the authenticity of this finding, five fragments of genes (V7S33_01155, V7S33_4525, V7S33_5150, V7S33_13785, V7S33_13965) were cloned and sequenced (Table 2). The BLAST results declared that V7S33_05150 could be regarded as an endoglucanase (EC 3.2.1.4) with 100% identity, while V7S33_13785 belonged to β-glucosidase (EC 3.2.1.21) in strain Z2.6 with 99.57% identities. Notably, V7S33_05150 was detected with a carbohydrate-binding site, which may enhance carbohydrate catalytic abilities [14,52], and V7S33_13965 also contained a signal peptide, suggesting a possible extracellular role in hydrolyzing (1->4)-beta-D-glucosidic linkages. These genes all exhibited similarities above 99.5% against sequences in other *B. velezensis* strains. Previous studies have elucidated the purified endoglucanases in *B. velezensis* with high activities [18,19], combined with the efficiency to cleave cellobiose by enriched β-glucosidase. This may ultimately empower superior lignocellulose degradation capabilities in *B. velezensis*. Thus, considering the annotations and validations, the genome of strain Z2.6 was abundant in cellulase and hemicellulose genes, substantiating its candidacy for lignocellulose utilization.

### 3.4. Process Optimization for Cellulase Production

#### 3.4.1. Optimum Enzymatic Condition

Determining enzymatic optimum conditions acted as a prerequisite for cellulase production optimization. The extracted crude cellulase was quantified utilizing the DNS method with reference to an absorbance standard curve (Figure S6a, $R^{2}$ > 0.99). By correlating the glucose-generated assays with the enzyme activities shown in Figure S6b–c, the optimum conditions of the crude cellulase mixture were determined to be 50 °C and pH 6.39. It is noteworthy that strain Z2.6 and other microorganism strains exhibited a common optimum temperature range (50 °C–60 °C), as evidenced by previous data [53,54]. This range also took effect for purified cellulase from *B. velezensis* [18,19]. Additionally, strain Z2.6 also performed a robust activity, maintaining above 75% in the range 40 °C–60 °C. For optimum pH, the determination of pH at 6.39 (Figure S6c) distinguished it from the commonly employed acid conditions of around 4.8 [28], but it intriguingly aligned with the optimal pH observed for the above-mentioned purified enzymes [18,19]. This outcome suggested that Z2.6 may possess an advantage in cellulose production efficiency. The collective discussion implies that the functional potentiality of cellulases from strain Z2.6 may diverge from that of other strains such as M2 [5] and ASN1 [53], which typically perform optimally under slightly acidic to neutral environments. Thereupon, the two determined conditions were applied in continuous process optimization.

#### 3.4.2. Effect of Independent Factors

**Figure 3:**
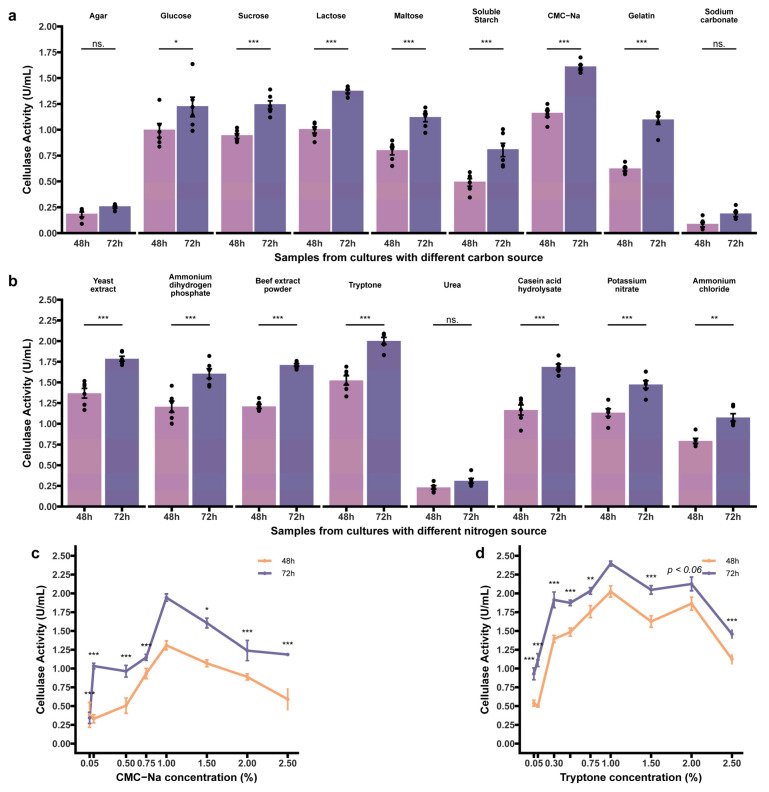
Effect of carbon and nitrogen sources and their concentrations on cellulase production. Effects on (**a**) carbon source; (**b**) nitrogen source; (**c**) CMC-Na concentration; (**d**) tryptone concentrations. Two-way ANOVA detected *p <* 0.001 for both effects of carbon and nitrogen sources, and further Tukey post hoc test individually revealed significant difference (*p* < 0.001, excluding tryptone–yeast extract *p* < 0.02) to reference groups as CMC-Na (**a**) and tryptone (**b**), and each source measured at two time points is annotated with codes. For (**c**,**d**), the chosen sources CMC-Na and tryptone illustrated the dynamics of activities with codes representing the comparison between each concentration to the point showing the highest activity. Significance codes are “***”, “**”, and “*”, representing *p* < 0.001, 0.01, and 0.05, respectively, while “ns.” means nonsignificant (*p*-values > 0.05). Data are presented as mean ± SEM.

With a foundational understanding of the capabilities in cellulose decomposition of strain Z2.6, we next shifted our sights towards elevating its production through a straightforward and accessible approach. Initial investigations were conducted using the single-factor-at-a-time methodology to facilitate incremental improvements, thereby supporting further optimization. In accordance with Figure 3, the cellulase activity of strain Z2.6 was first assessed in relation to the carbon and nitrogen sources in the medium. Cultures supplemented with CMC-Na demonstrated the greatest activities at both 48 h and 72 h (1.16 U/mL and 1.61 U/mL, respectively), accompanied by *p*-values compared to CMC-Na being all <0.001. This significant increase underscored the efficiency of CMC-Na in enhancing cellulase activity. This observation may be attributed, at least in part, to CMC-Na facilitating predominant endoglucanase and β-glucanase activities [19,55]. Nevertheless, carbon sources, such as agar or inorganic sodium carbonate, in both time scales, exhibited minimal to negligible activities (<0.3 U/mL), suggesting their inefficient utilization. Among the tested carbohydrates, including sucrose, lactose, and maltose, higher cellulase activities were evident compared to gelatin and soluble starch, indicating their possible suitability in generalized optimization studies [56]. Consequently, CMC-Na emerged as the optimized carbon source. Moreover, a detailed investigation of carbon source concentration is illustrated in Figure 3c. A concentration of 1.0% (*w*/*v*) exhibited a peak, signifying advantages over other concentrations. Low concentrations (<0.50%) appeared insufficient for effective cellulase production (Figure 3c), whereas colloidal environments induced by increasing CMC-Na to high concentrations might restrict the growth of bacteria [57,58], thereby impairing cellulase production. Hence, the independent contribution of CMC-Na was confirmed to be maximal at 1.0%, retaining its essential role in the medium.

The operations concerning nitrogen sources were elucidated as depicted in Figure 3b. Categorized into organic and inorganic, their comparisons revealed that, excluding urea, inorganic sources were holistically weaker than organic sources, a phenomenon in agreement with Djelid et al. [18], which is commonly inclined to show conspicuous activities in the presence of organic nitrogen sources. Furthermore, peptone, yeast extract, and tryptone demonstrated similar performance levels, each possessing distinct advantages in different research applications [3,18,59]. Minimal deviations were observed in yeast extract, beef extract powder, and casein acid hydrolysate. Conversely, urea processing performed the poorest, with negligible activity, corroborating findings from the urease test in the biochemical analyses (Table 1). Among the nitrogen salt tested, NH_4_H_2_PO_4_, KNO_3_, and NH_4_Cl displayed relatively normal activities, although NH_4_Cl utilization underperformed. These comparisons between organic and inorganic nitrogen sources underscore the indispensability of organic nitrogen sources for superior cellulase production [3], even in scenarios where the impact of inorganic sources might be mitigated, likely due to compensatory responses stemming from the original optimal carbon source level. Notably, tryptone exhibited the highest activity, averaging 1.52 U/mL and 2.00 U/mL at 48 h and 72 h, respectively, positioning it as the optimal selection. For the examination of tryptone concentration ranging 0.05–2.5%, the effect of the concentration of 1.0% (*w*/*v*) produced activities exceeding 2.0 U/mL (Figure 3d). The utilization of tryptone was also adopted by previous optimization of cellulase production in the *Bacillus* sp. [60,61]. Consequently, tryptone was ultimately chosen as the nitrogen source, with an initial concentration of 0.3% in FM broth, paving the way for the following optimization.

**Figure 4:**
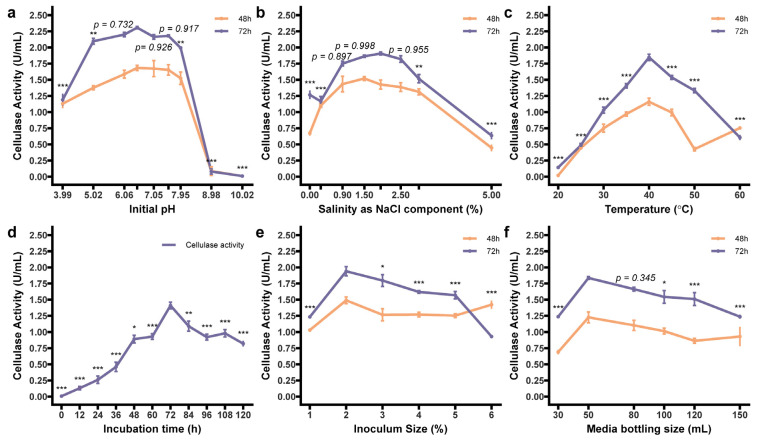
The rest of the one-factor-at-a-time design that induces outstanding crude cellulase activity. The results measured here were produced by FM broth with 1.0% (*w*/*v*) CMC-Na as carbon source and 0.3% (*w*/*v*) tryptone as nitrogen source. (**a**) Initial pH; (**b**) temperature (°C); (**c**) salinity (%); (**d**) time (h); (**e**) inoculum size (%); (**f**) media bottling volume (mL). All six factors, by two-way ANOVA, provided effects on cellulase production, and the Tukey post hoc test is marked with codes for other measurements against the reference group. Significance codes are “***”, “**”, and “*”, representing *p* < 0.001, 0.01, and 0.05, respectively, with nonsignificant ones showing specific *p*-values. Data are presented as mean ± SEM.

After the determination of the above dominant sources, we also intended to enrich the comprehension of single-factor optimization for strain Z2.6. Cellulase activity reached its zenith at pH 6.49, with its surrounding range (pH 6.0–7.5, *p* > 0.73) yielding unobvious attenuation in enzyme activities, and robustness was clarified by at least > 86% capability performing from 5.02 to 7.98 (Figure 4a). This wide adaptability covered previous studies with optimized pH of either acid or alkalescent environment [3,5,53,62], indicating a potential to treat the extensive raw materials of *B. velezensis*. A slight limitation was shown in extreme initial pH, where the activity nearly disappeared at alkaline pH 10.02. For the salinity study, NaCl was additionally added to the medium. Strain Z2.6 showed strong adaptability with the exception of extremely high concentrations, such as 5.0% NaCl, where merely about 34% relative activity was maintained. Meanwhile, at 0% NaCl, Z2.6 may need an accumulation in time to demonstrate relatively high activity (Figure 4b). The peak emerged as 1.95% NaCl, at which previous and subsequent fluctuations in salinity resulted in a decrease in activity to a small extent. Although this would contradict optimal NaCl concentration for endoglucanase activity with a superior halotolerance [18], it was still located in an efficient range, and the high moisture caused by the salinity increasing may act on bacteria growth and cellulase production. Then, we moved on to cultivation temperature, and a spurt to 40 °C at 72 h (1.85 U/mL) made it a superior choice for fermentation, and an optimum temperature of 40 °C was also reported for the *Bacillus* sp. [63] and the cellulase of *B. velezensis* [19]. The performance in the range 25–60 °C reversed >40% of activities with a quick impact at higher temperatures around 60 °C (Figure 4c), which allowed it to play a vital role in cellulose utilization in the whole composting.

With the progression of fermentation, a rapid ascent in cellulase activity was observed from 24 h to 72 h, reaching a peak of 1.41 U/mL, followed by a stabilization phase where activity levels hovered at 71% beyond 84 h (Figure 4d). This peak at 72 h aligned with findings reported for cellulase production and purified cellulases from *B. velezensis* [18,19]. Additionally, preponderances were shown when extending fermentation time to 72 h, indicating careful control of fermentation duration was warranted to maximize benefits in cellulase production. The next stage primarily focused on the remaining two parameters, which have received comparatively less attention. The highest cellulase activities were attained with an inoculum size of 2% and a media bottling volume of 50 mL, resembling conditions reported for *B. velezensis* M2 isolated from piglet manure [5]. Overall variation in inoculum size showed robust effects with the highest activity at 2% scale after 72 h cultivation. On the contrary, lower inoculum sizes, such as 1%, were in relatively low activity levels, while a convergence of two lines was presented between the 5% and 6% scales (Figure 4e). It is essential to note that excessively high inoculation size may lead to an excessive bacteria density, accelerating the consumption of dissolved oxygen and nutrients in the medium, finally creating an inadequate environment detrimental to enzyme-producing ability. Regarding media bottling volume, a slight advantage was shown with a volume of 50 mL (1.84 U/mL), while subsequent volume increases led to fluctuations in activity (Figure 4f). Although a volume of 80 mL exhibited no significant difference, this range conformed to *B. velezensis* Y1 [19]. In our study, the optimum inoculum size and bottling volume were determined to be 2% and 50 mL, respectively, pledging an appropriate liquid loading to provide appropriate aqueous nutrients [64]. These conditions were further used for preliminary experiments and subsequent exploration of dominant factor screening. Thus, the determination of optimal single parameters through the aforementioned trials facilitated efficient cellulase production in strain Z2.6 and provided valuable insights for future studies of their co-influences.

### 3.5. Statistical Optimization of Cellulase Production

#### 3.5.1. Independent Factors as Main Effects

Following the completion of the eight parameters confirmed using the one-factor-at-a-time design, we were next inclined to figure out predominant factors exerting higher contributions to cellulase production. Recent investigations on *B. velezensis* mainly centered on the isolation, characterization, and optimization of single fermentation conditions at one time [5,14,18,19], while a limited number of studies extended their analyses to incorporate response surfaces aimed at more efficient cellulase production [3,53]. To find the three most key factors among the eight factors, of which the carbon and nitrogen source were already affirmed as CMC-Na and tryptone, a PB design was used for this duty, shown in Table 3, where the response results are shown. A total of eight variables, namely CMC-Na (A), tryptone (B), temperature (C), initial pH (D), salinity (E), inoculum size (F), media bottling size (G), and incubation time (H), and the remaining three dummy variables (I, J, K), which were not expected to contribute to the model, were examined by 12 runs in the PB design. Next, the main effect of each variable upon cellulase production was estimated as the difference between both averages of measurement made at the low level (−1) and the high level (+1) of that specific factor.

The model structure was subjected to significance testing (*p <* 0.005), as implied by ANOVA correlating each run (Table S8). Components or fermentation conditions were assessed within a confidence interval, along with their corresponding *p*-values. The results allowed the prediction of positive or negative effects, suggesting requirements of high or low value changes [65], where types of effects for Z2.6 are presented as signs in Equation (1) as either plus (+) or minus (−). According to this assay, CMC-Na, salinity, and tryptone all presented *p <* 0.02, demonstrating significance and warranting their selection (Table S8). Meanwhile, the dummy variables exhibited nonsignificance, with the distributions of their interactions (AB, AC, and AG) all below 5%. Apart from the dummy variables, only temperature and pH exerted negative effects, with temperature contributing more towards cellulase production. This observation was in agreement with reports from Nair et al. [53] and Sherief et al. [66], albeit at lower levels in this study. Furthermore, we elicited another factor, salinity, which exhibited substantial contributions and supported the positive correlation between NaCl concentration and cellulase production, as previously reported by Singh et al. [67].

Therefore, a linear regression model was established based on the experimental data, with quality indicated by the determination coefficient, R^2^. In total, three kinds of R^2^ were evaluated, in which the obtained R^2^ value was 0.8643, indicating that 86.43% of the variability could be explained. Moreover, the value of the adjusted determination coefficient (adj R^2^) was presented as 0.8134, with the distance to predicted R^2^ less than 0.2, symbolizing high significance for the model. Moreover, an adequate signal of 12.009 was also measured, with “Adeq Precision” greater than 4. Therefore, the three significant variables were chosen as CMC-Na, tryptone, and salinity, forming Equation (1) as follows:

Y_PB_ = 1.7498A + 1.0066B + 0.3871E − 0.6575 (1)

where A, B, and E represent the same variables in Table 3, and their levels should be specified in original units instead of coding as +1 or −1. After affirmation of the three most significant effects on cellulase production, all other variables in continuous trials were kept to the optimum level analyzed before.

#### 3.5.2. Path of Steepest Ascent Design

To confirm a pivotal point for ongoing optimization, the steepest ascent path design was executed. Three significant variables were adjusted steeply: CMC-Na and tryptone concentrations were incremented by 0.25%, while salinity was increased by 0.45%. All variables exhibited an upward trend, with other factors maintained at their optimal levels (Table 4). The direction of ascent was determined by statistical results directly, while the step size also took single-factor experiment data into consideration, thus narrowing the parameter range for RSM [41,68]. Observations revealed that cellulase activity climbed from run 1 to 3 but began to decline at run 4. Of note, run 3 showed the highest cellulase activity of 2.76 ± 0.04 U/mL, serving as the peak. In this run, the three key factors were 1.0% CMC-Na, 1.95% NaCl, and 1.0% tryptone in the medium. Consequently, this option, situated near the region of maximum activity, was chosen for the BBD design with a considerable range surrounding the point.

#### 3.5.3. Response Surface Method by Box–Behnken Design

Given the emerging key factors and pivotal point, our attention shifted towards their synergistic integration using response surface. RSM was employed to ascertain the optimal level of parameters derived from the PB design and to elucidate the intricate interactions among these factors, namely CMC-Na (X_1_), salinity (X_2_), and tryptone (X_3_). The BBD matrix, structured around the center point informed by the above steepest ascent test, incorporated coding levels and responses across 17 combinations, as presented in Table 5. Moreover, the ANOVA test (Table S9) results revealed an *F*-value of 9.14, indicating only a 0.40% chance it could occur from noise. This underscored the statistical significance of fitting a quadratic mode. Additional scrutiny of the model unveiled a “lack-of-fit *F*-value” of 5.8, accompanied by an insignificant *p*-value, suggesting the robust fitness was hardly disrupted by incidental noise. Further, adequate precision was the measurement of the ratio of signal to noise, and the resulting value of 8.06, above the cutoff of 4.0, could be a preference for the model.

In this model term, four quadratic (X_1_^2^, X_2_^2^, X_1_X_2_, and X_2_X_3_) terms and one linear (X_1_) term were recognized as significant sources, in which X_1_^2^ and X_2_^2^ were of more visible significance (*p* ≤ 0.018), providing better correlations and contributions to response activities. Yet, tryptone seemed to have lower performance relative to cellulase production (*p* = 0.058), which was only significant in terms of the interaction of X_2_X_3_. By the following mathematical analysis, a multiple regression equation was generated from those data, with combined influence from the factors. The obtained model interpreting the relationship within key factors corresponding to cellulase production (Y_BBD_) was constructed with the second-order polynomial Equation (2) below:

Y_BBD_ = 2.83 + 0.1533X_1_ + 0.1224X_2_ + 0.1370X_3_ + 0.2121X_1_X_2_ + 0.0737X_1_X_3_ + 0.2105X_2_X_3_ − 0.4086X_1_^2^ − 0.2057X_2_^2^ − 0.105X_3_^2^ (2)

where X_1_, X_2_, and X_3_ represent CMC-Na, salinity, and tryptone, respectively. The determination coefficient, R^2^, was calculated as 92.16% for this regression, with the adjusted determination coefficient being 82.08%, clarifying the consistencies between practical and predicted cellulase production. This alignment in the two R^2^ values provided a good estimation of the cellulase production responses within the range of the process conditions [41]. On this basis, the effects of substrates and conditions and their interactions on the responses of the cellulase production were further visualized.

**Figure 5:**
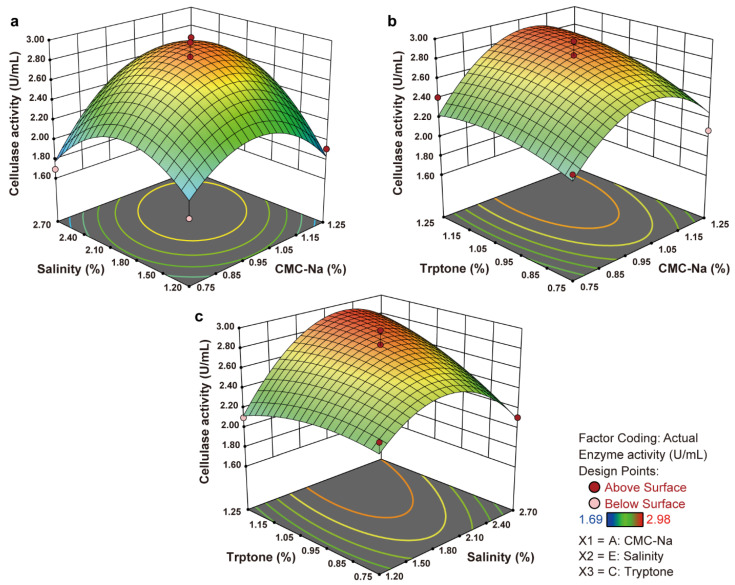
Response surfaces for optimizing cellulase production by BBD. Three 3D response surfaces for cellulase production showing the interactive effects of actual medium components as CMC-Na (X_1_), salinity (X_2_), and tryptone (X_3_) levels as: (**a**) X_1_–X_2_ interaction; (**b**) X_1_–X_3_ interaction; (**c**) X_2_–X_3_ interaction. Legend contains different representatives for each point, gradient ramp, and variable name. A range of 1.69 U/mL to 2.98 U/mL was reflected from the gradient ramp by the response surfaces, with the relationship of each experimental point and the regressed surface.

The responsiveness of the factors, both individually and synergistically, for cultivating *B. velezensis* Z2.6 are illustrated with 3D response curves in Figure 5. Each plot depicts the effects of two separate variables while keeping the remaining one constant in detail as the interactions between CMC-Na and salinity (X_1_–X_2_, Figure 5a), CMC-Na and tryptone (X_1_–X_3_, Figure 5b), and salinity and tryptone (X_2_–X_3_, Figure 5c). The surface in Figure 5a displays visible convexity, with projections in the bottom panel appearing almost circular, suggesting a minor interacting influence on cellulase production. However, Figure 5b–c show two elliptical contours on the convex response surfaces, revealing that interactions involving salinity and other factors could not be overlooked. Sketchy axis adjustments were made to track factor values for the maximum production [69], reflecting the highest response at approximate concentrations of 1.08% CMC-Na, 2.15% salinity, and 1.22% tryptone for strain Z2.6. Enhancements in cellulase production were observed with increases in any tree factors within reasonable ranges prior to the above estimations.

The maximum production values were predicted through an integration of the above numeric and graphical methodologies, yielding an optimal solution for cellulase response given by 1.09% CMC-Na, 2.30% salinity, and 1.23% tryptone, resulting in a predicted maximum cellulase activity of 2.98 U/mL. Experimental validation under these optimal incubation conditions, in triplicate, yielded a final cellulase activity reaching 3.02 ± 0.03 U/mL, elucidating approximately a 3.43-fold optimization compared to the initial non-optimized production and verifying the adequacy of the model. In summary, the precise optimization of cellulase production in strain Z2.6 demonstrated the efficiency and efficacy of RSM in optimizing medium formulation for enzyme or product yields [41,53]. The optimized medium in this study was (*w*/*v*) 1.09% CMC-Na, 1.23% tryptone, and 2.30% salinity, with other parameters in optimal levels after 72 h incubation. CMC-Na was an inducer of cellulase production due to its high contribution and significance, consistent with reports by Deka et al. [58]. Salinity, another predominant factor, should also be emphasized due to its own effects and interactions with the other two factors in this study. Additionally, the carbon-to-nitrogen ratio of 1.13 utilized in this medium closely resembled the optimal ratio 1.0 for maximal activity of β-glucanase reported by Khalid et al. [19], suggesting a cooperation in building a conducive circumstance. These results provide valuable recommendations for further cellulase production in *Bacillus* sp. and potential applications in industrial fermentation manufacturing processes.

*B. velezensis* Z2.6, isolated from compost, is a heterotrophic microorganism with cellulose utilization capability, as comprehensively elucidated in this study through the examination of its fundamental parameters in crude cellulase application. Initial screening of cellulase-producing candidates revealed conspicuous transparent zones on selective media, along with considerable enzymatic activity without optimization, prompting our investigation. An early report by Peixoto et al. [70] had already noticed the potential cellulase production of *B. velezensis*, indicating an initial basal level of cellulase activity prior to optimization [3,5]. Given the successful application of statistical design techniques such as RSM in optimizing enzyme production, including cellulase, protease, and uricase in various *Bacillus* species [58,61,65], we further employed exquisite BBD, a specific RSM approach signifying the promotion process [71], to interpret the inherent potentialities in cellulase production. Therefore, meticulous progressive optimization contributed superior increases in cellulase yield, merely through carefully altering the medium composition, reaching a final optimum more than 3.4-fold higher than the initial baseline, which enriched the research in developing cellulase production in *B. velezensis*. Previous studies on *B. velezensis* with RSM reported similar enhancement degrees, with a 3-fold increase for strain ASN1 [53] and a 3.3-fold increase for strain A-68 [3], both slightly weaker than strain Z2.6. While some studies have touched upon optimization, they may not have pursued comprehensive optimization, as undertaken in this study. The validated cellulase production of strain Z2.6 (3.02 U/mL) surpassed that of strain ASN-1 (2.42 U/mL). Simultaneously, other post-optimal cellulase activities such as *B. subtilis* M-11 (0.43 U/mL), *B. halodurans* IND18 (4.14 U/mg), and *B.* VITRKHB (1.92 IU/mL) [63,64,66] indicated that strain Z2.6 showed a cross-species competitive capability in cellulose degradation. These findings collectively underscored the efficacy of RSM in precisely predicting optimal conditions for enhancing cellulase yields, offering eco-friendly alternatives to traditional chemical methods for processing cellulose materials [72].

In efforts to maximize cellulolytic bacteria output straightforwardly by alternating media components, major studies have focused on substrates, pH, and temperature in *Bacillus* sp. cultivation, but a comprehensive integrated description of individual and cooperative effects for each factor in the media components was absent for *B. velezensis*. Strain Z2.6 has streamlined this approach by using common reagents that theoretically offered versatility for cellulosic materials in pre-treatment scenarios. Of paramount importance, carbon sources containing carboxymethylcellulose performed remarkably positively in inducing cellulase production [58,72,73]. Specifically, CMC-Na navigated 6.81-fold and 3-fold increases in species *B. amyloliquefaciens* MBAA3 and *B. licheniformis* NCIM5556, respectively, and was also favored by strain Z2.6 with overall significance [65,74]. As mentioned, similarities co-embodied in individual strains using nitrogen sources are commonly acknowledged for their positive effects on cellulase production, such as tryptone, peptone, and yeast extract [3,18,59,63], indicating a non-stringent demand on nitrogen sources. This study further emphasized the significance of moderate salinity in influencing factorial interactions that contributed to production, which caused less attention unless in studies with a focus on halotolerance [43,52]. The preference of such salinity (approximately 2% NaCl) could be partially attributed to the saline-abundant conditions in composting due to both animal excrement and marine waste. Of note, two major cellulases, endoglucanase and β-glucosidase, consistent with previous purified ones [13,25], were detected among the five cloned gene fragments of Z2.6, supporting practical prospects for its crude cellulase mixture. Not only did these cellulases exhibit analogous levels of suitable enzymatic conditions, such as 50 °C–60 °C and pH below neutral, the inducing environment in the media also concurred with fermentation temperature, time, and liquid loading. In consolidating media compositions, similitude was described in the optimization strategies for bacteria such as *B. subtilis* AS3 (utilizing carboxymethylcellulose, NaCl, MgSO_4_, and (NH_4_)_2_SO_4_) [58] and *Enhydrobacter* sp. ACCA2 (utilizing carboxymethylcellulose, peptone, MgSO_4_, and K_2_HPO_4_) [72], both of which demonstrated over a 2.3-fold ascent in cellulase production. Additionally, strain Z2.6 exhibited acceptable resistance to acidity (pH 4.0) and heat (60 °C), retaining approximately 59% and 50% activity, respectively. These characteristics rendered it suitable for a wide range of applications in cellulose decomposition. In conclusion, strain Z2.6 succeeded in the optimization of cellulose-degrading capabilities, attempting to establish a preliminary guidance to reach such outstanding cellulase activities. The high-level cellulase production and adaptability to adverse conditions laid a solid foundation for economical and efficient bacterial usage. While it has been established that the genome of strain Z2.6 coded for the two major cellulolytic enzymes, there remain limitations in regarding the crude cellulase mixture as the research object. Therefore, a more specific collaboration involving purified enzymes could be further considered. Nonetheless, it is certain that through this study, a further excavation of secondary biomass usage, such as the compost of algae residuals, for microorganism resources and a comprehensive understanding of *B. velezensis* Z2.6 have been achieved. This study might provide insights for directly obtaining an efficient lignocellulose degradation for industrial pre-disposal or the novel study of similar species. Combining the findings in this article with other previous and forthcoming multilevel studies, the challenge of reducing cellulase production costs could be ultimately addressed, promising to unlock sustainable applications for cellulolytic bacteria.

## 4. Conclusions

This study isolated and characterized a bacterium, *B. velezensis* Z2.6 (=CGMCC 28578), with lignocellulolytic effect and saccharification potential from pig manure-based compost with algae residuals as feedstock. The Gram-positive, rod-shaped cells formed creamy white, rough, minorly translucent colonies. Growth occurred in the presence of 0–8% NaCl, temperature 10 °C–60 °C, and at least pH 4.0–9.0, simultaneously showing optimal conditions for the crude enzyme at pH 6.49 and 50 °C. The GenBank accession number deposited for the draft genome sequence of strain Z2.6 is JBANFQ000000000. As a potential novel strain for cellulase production, strain Z2.6 was detected with 31 fragments of cellulolytic-related genes, including endo-1,4-β-D-glucanase (EC 3.2.1.4) and β-glucosidase (EC 3.2.1.21). The enzyme mixture underwent comprehensive optimization, which consisted of a single-factor-at-a-time completed design and statistical processing. Among the ten explored factors, CMC-Na, salinity, and tryptone were recognized as key factors, whose interactions were further investigated by BBD with 3D response surfaces. The final optimized results elucidated a remarkable 3.43-fold increase in cellulose production. This meticulous analysis of optimal conditions for cellulase production encompassed fundamental variables, providing valuable insights that can serve as navigation for the quantitative production of cellulases. In summary, this exploration of strain Z2.6 may enlighten the prospect of utilizing compost with forward marine raw materials for bacterial cellulase production, suggesting the intensive reuse value of bioproducts and biowaste. Regarding full qualities and functionalities, *B. velezensis* Z2.6 stands as a competitive and cost-effective candidate to meet the requirements of cellulose biodegradation.

## Figures and Tables

**Table 1:** Physiological and biochemical characteristic features.

Characteristic	Results ^1^	Characteristic	Results ^1^
Lactose	+	Catalase	+
Sucrose	+	Urea	−
Glycerol	+	Lysine	−
Mannose	+	Arginine	−
Sorbitol	−	Ornithine	−
Salicin	−	Indole	−
Dulcitol	−	VP test	−
Esculin	+	Methyl red test	−
Raffinose	−	6% NaCl	+
ONPG	+	8% NaCl	+
Gelatin	+	10% NaCl	−
Propionate	−	pH 6.0	+
Malonate	−	pH 5.0	+
Simon’s citrate salt	−	Growth at 10 °C	+
Amylolysis	+	Growth at 70 °C	−

^1^ Symbols in results: +, positive; −, negative.

**Table 2:** Cloning of representative cellulases annotated by CAZy database from *B. velezensis Z2.6*.

Locus Accession Number	CAZy Family	Annotation (EC Codes)	CBM Family ^1^	Signal Peptide	Most Identical Sequence to *B. velezensis* (% Identity)	Most Identical Sequence to Non-*bacillus* [Species] (% Identity)
V7S33_01155	GH1	Alpha-N-arabinofuranosidase (EC 3.2.1.55)	− ^2^	−	WP_326142774.1 99.80%	P45797.1 [*Paenibacillus polymyxa*] 75.46%
V7S33_04525	GH1	6-phospho-beta-glucosidase (EC 3.2.1.86)	−	−	WP_129091804.1 99.39%	Q9KBR4.1 [*Halalkalibacterium halodurans* C-125] 73.03%
V7S33_05150	GH5 subfamily 2	Endoglucanase (EC 3.2.1.4)	CBM3	−	WP_032875077.1 100%	Q46829.2 [*Escherichia coli* K-12] 65.40%
V7S33_13785	GH1	Beta-glucosidase (EC 3.2.1.21)	−	−	WP_285980062.1 99.57%	Q47096.1 [*Pectobacterium carotovorum* subsp. *carotovorum*] 53.50%
V7S33_13965	GH16 subfamily 21	Beta-glucanase (EC 3.2.1.73)	−	Yes	WP_308826282.1 99.59%	P26208.1 [*Acetivibrio thermocellus* ATCC 27405] 38.48%

^1^ CBM is the abbreviation for Carbohydrate Binding Module. ^2^ “−” in the table indicates no corresponding results were detected.

**Table 3:** Plackett–Burman design in real values for eight variables. Responses were calculated by the average of triplicate as mean (SD).

Run Order	Variables ^1^								Response Cellulase Activity
	A	B	C	D	E	F	G	H	
1	0.5	1	45	6.5	0	2	50	72	1.177 ± 0.08
2	0.5	0.5	40	6	0	2	50	48	0.756 ± 0.07
3	0.5	0.5	45	6	1.5	4	50	72	1.229 ± 0.08
4	1	1	40	6	0	4	50	72	2.167 ± 0.11
5	1	0.5	45	6.5	1.5	2	50	48	1.694 ± 0.06
6	1	1	45	6	0	2	80	48	1.947 ± 0.04
7	0.5	0.5	40	6.5	0	4	80	48	0.535 ± 0.06
8	0.5	1	40	6.5	1.5	2	80	72	2.003 ± 0.04
9	0.5	1	45	6	1.5	4	80	48	1.876 ± 0.10
10	1	0.5	45	6.5	0	4	80	72	1.976 ± 0.06
11	1	1	40	6.5	1.5	4	50	48	2.642 ± 0.05
12	1	0.5	40	6	1.5	2	80	72	2.499 ± 0.09

^1^ Letters in “variables” represented as A: CMC-Na (%); B: tryptone (%); C: temperature (°C); D: pH; E: salinity (%); F: inoculum size (%); G: media bottling volume (%); H: time (h). Remaining 3 variables, I, J, and K, are assumed as dummy variables.

**Table 4:** Cellulase activity results along the path of steepest ascent. Responses were calculated by the average of triplicate as mean (SD).

Run	CMC-Na (%)	Salinity (%)	Tryptone (%)	Cellulase Activity (U/mL)
1	0.50	1.05	0.50	0.891 ± 0.03
2	0.75	1.50	0.75	1.678 ± 0.08
3	1.00	1.95	1.00	2.764 ± 0.04
4	1.25	2.40	1.25	2.509 ± 0.05
5	1.50	2.85	1.50	1.988 ± 0.11

**Table 5:** The Box–Behnken design matrix for exploring effects and coeffects, along with responses. Three dominant variables were CMC-Na, salinity, and tryptone. Responses were calculated by the average of triplicate as mean (SD).

Run Order	Variable			Response: Enzyme Activity (U/mL)
	Factor 1: CMC-Na (%)	Factor 2: Salinity (%)	Factor 3: Tryptone (%)	
1	1	0.65	1	2.979 ± 0.10
2	1	0.9	1.25	2.685 ± 0.11
3	1	0.4	1.25	2.106 ± 0.09
4	1.25	0.65	0.75	2.076 ± 0.06
5	1.25	0.65	1.25	2.621 ± 0.14
6	1	0.65	1	2.814 ± 0.08
7	1	0.9	0.75	2.114 ± 0.03
8	0.75	0.65	0.75	2.162 ± 0.07
9	1.25	0.9	1	2.671 ± 0.05
10	1	0.65	1	2.705 ± 0.07
11	1	0.4	0.75	2.377 ± 0.04
12	0.75	0.9	1	1.695 ± 0.05
13	1	0.65	1	2.819 ± 0.05
14	1	0.65	1	2.840 ± 0.09
15	0.75	0.65	1.25	2.413 ± 0.10
16	0.75	0.4	1	1.787 ± 0.02
17	1.25	0.4	1	1.916 ± 0.06

## Data Availability

This draft genome of strain Z2.6 was deposited at DDBJ/ENA/GenBank under the accession number JBANFQ000000000, and the version described in this paper was version JBANFQ010000000. Other original contributions presented in this study were included in the article and the Supplementary Materials. Further inquiries can be directed to the corresponding authors.

## Supplementary Materials

The following supporting information can be downloaded at: https://www.mdpi.com/article/10.3390/microorganisms12050979/s1, Figure S1: Medium selection with morphological observation and growth curve of strain Z2.6. (a) Isolation of Z2.6 on CMC-Na agar (CA) with streak plate techniques at 24 h. (b) Spot incubation of Z2.6 after 48 h followed by Congo-red stain in (e). (c) Gram staining of Z2.6 that the blue-violet color of colonies is illustrated after re-staining and elution (100×). (d–f) represent spot-incubated Z2.6 with Congo-red staining at 24 h, 48 h, and 72 h, respectively. (g) Overall growth data are shown and regressed. Observed bacteria densities (OD600nm) are presented as bars in yellow with standard deviation standard as error bars. Based on the logistic S-curve, points are predicted by the “deSolve” package with an ordinary differential equation, presenting a significant well-fitting regression; Figure S2: The type (strain) genome sever (TYGS) tree based on genome BLAST distance phylogeny (GBDP). Branch lengths are scaled in terms of GBDP distance presented in red color, while blue numbers above branches are GBDP pseudo-bootstrap support values from 100 replications; Figure S3: The gene function prediction based on the database of clusters of orthologous (COG) genes. The COG functions are labeled by A-Z on the X-axis and interpreted in the right legend followed by numbers in brackets. The numbers are also presented above each bar in specific colors; Figure S4: Statistical map of gene clusters and number of corresponding genes for strain Z2.6. Predicted by antiSMASH 4.0.2 software, 11 kinds of secondary metabolites were shown. Each cluster was labeled with red and blue for cluster number and gene number in this cluster; Figure S5: Functional classification map based on the CAZy annotation. Statistical map with the number of genes belonging to the six classification notes based on the CAZy database. Above is the sample ID and the horizontal coordinate is the corresponding classification type, namely Auxiliary Activities (AAs), Carbohydrate-Binding Modules (CBMs), carbohydrate esterases (CEs), glycoside hydrolases (GHs), glycosyl transferases (GTs), and polysaccharide lyases (PLs); Figure S6: Enzymatic reaction optimum conditions of crude cellulase. A standard curve (a) was regressed by absorbances (OD_540nm_) versus glucose concentration as the reference in the DNS method, with a determination coefficient (R^2^) greater than 0.999. Glucose concentration was diluted from the glucose standard solution (1.0 mg/mL). Optimal enzymatic conditions of (b) temperature and (c) pH indicate that optimum temperature and pH for the reaction are approximately 50 °C and 6.49 respectively. Data are all mean ± SEM in triplicates at each point; Table S1: Premiers used to clone cellulase-related genes in this study; Table S2: Details of complete randomized design in progressive one-factor-at-a-time screening; Table S3: Parameters and their two levels in PB design. Dummy means dummy variables, set to meet the experimental requirements; Table S4: Variables and their levels employed in BBD; Table S5: Spot inoculation with replicas assay for detecting transparent zone. Responses are calculated by the average of triplicate with standard deviation; Table S6: Summary of growth curve by nonlinear least square (NLS) in R; Table S7: Annotated genes encoding cellulose-degradation related enzymes of *Bacillus velezensis* Z2.6 by the CAZy database. “EC#” means the EC recording numbers; Table S8: ANOVA and model evaluation for Plackett–Burman design. Significance codes are 0.05 ‘*’, 0.01 ‘**’, and 0.001 ‘***’, where statistical signification is at the 95% confidence level (*p* < 0.05); Table S9: Responses according to the Box–Behnken method with the summary of ANOVA and model fitness. Significance codes are 0.05 “*” and 0.01 “**”.
