# Supplementary material for: Introduction of Cellulolytic Bacterium Bacillus velezensis Z2.6 and Its Cellulase Production Optimization
Source: Microorganisms. 2024 May 13;12(5):979. doi: 10.3390/microorganisms12050979 (PMC11124521; doi:10.3390/microorganisms12050979)
Supplement: Supplementary file 1 [file microorganisms-12-00979-s001.zip › microorganisms-2964939-supplementary.pdf]

Supplementary materials

Supplementary figures

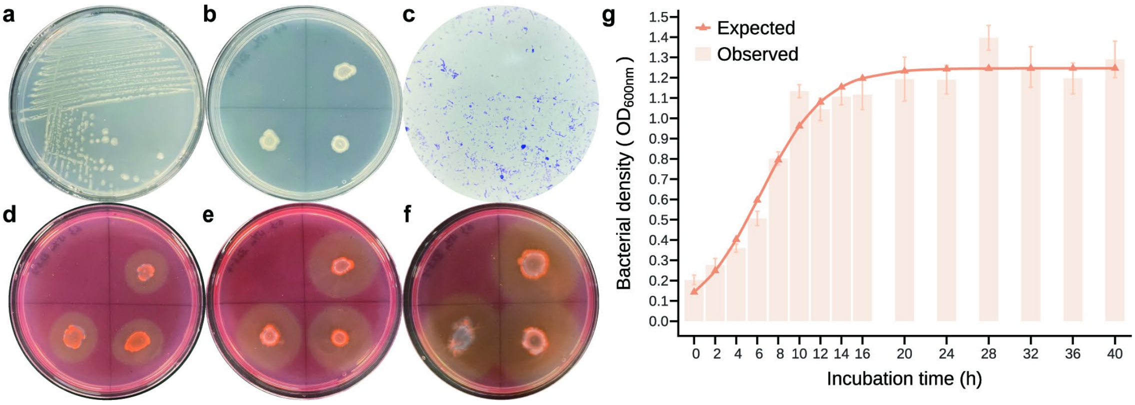

Figure S1. Medium selection with morphological observation and growth curve of strain Z2.6. **(a)** Isolation of Z2.6 on CMC-Na agar (CA) with streak plate techniques at 24 h. **(b)** Spot incubation of Z2.6 after 48 h followed by Congo-red stain in **(e)**. **(c)** Gram staining of Z2.6 that the blue-violet color of colonies is illustrated after re-staining and elution (100×). **(d–f)** represent spot-incubated Z2.6 with Congo-red staining at 24 h, 48 h, and 72 h, respectively. **(g)** Overall growth data are shown and regressed. Observed bacteria densities (OD_{600nm}) are presented as bars in yellow with standard deviation standard as error bars. Based on the logistic S-curve, points are predicted by the "deSolve" package with an ordinary differential equation, presenting a significant well-fitting regression.

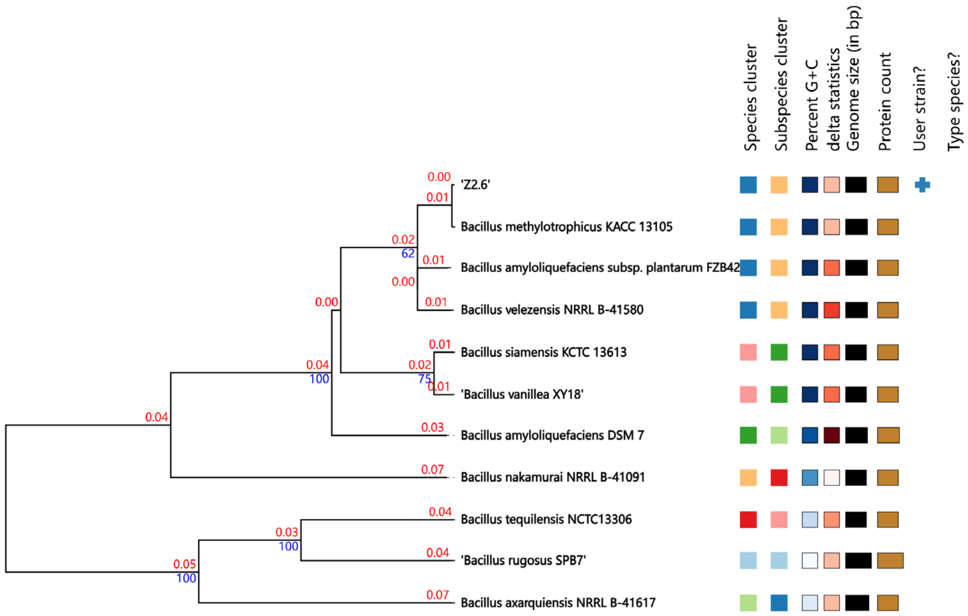

Figure S2. The type (strain) genome sever (TYGS) tree based on genome BLAST distance phylogeny (GBDP). Branch lengths are scaled in terms of GBDP distance presented in red color, while blue numbers above branches are GBDP pseudo-bootstrap support values from 100 replications.

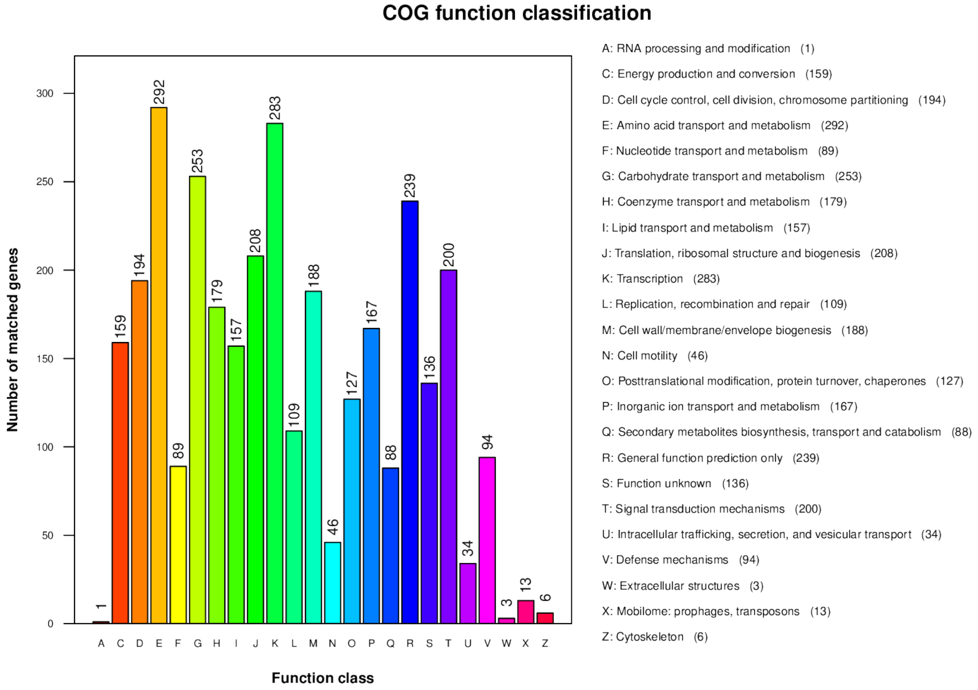

Figure S3. The gene function prediction based on the database of clusters of orthologous (COG) genes. The COG functions are labeled by A-Z on the X-axis and interpreted in the right legend followed by numbers in brackets. The numbers are also presented above each bar in specific colors.

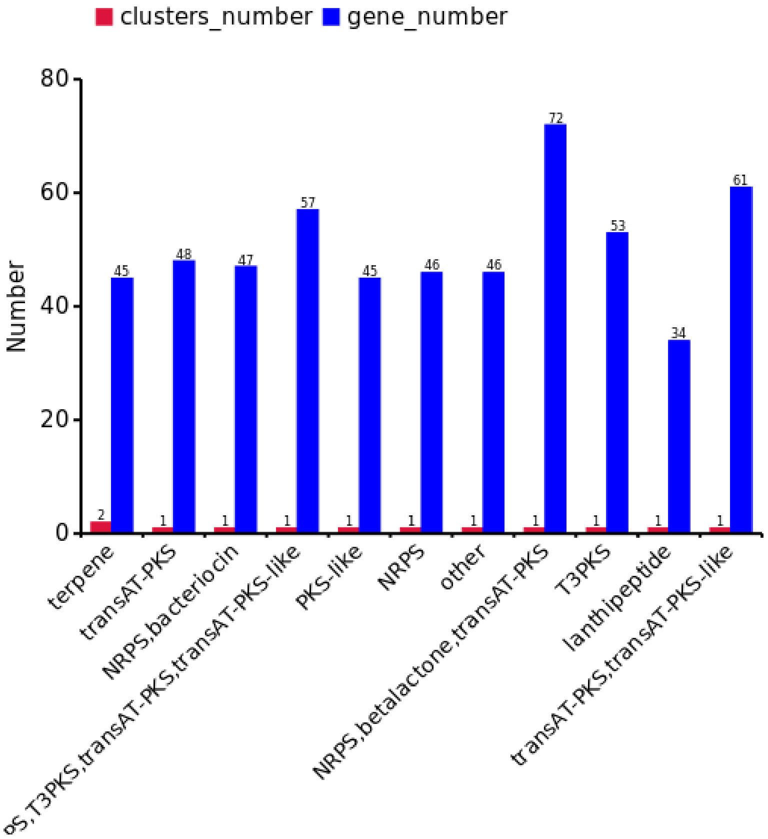

Figure S4. Statistical map of gene clusters and number of corresponding genes for strain Z2.6. Predicted by antiSMASH 4.0.2 software, 11 kinds of secondary metabolites were shown. Each cluster was labeled with red and blue for cluster number and gene number in this cluster.

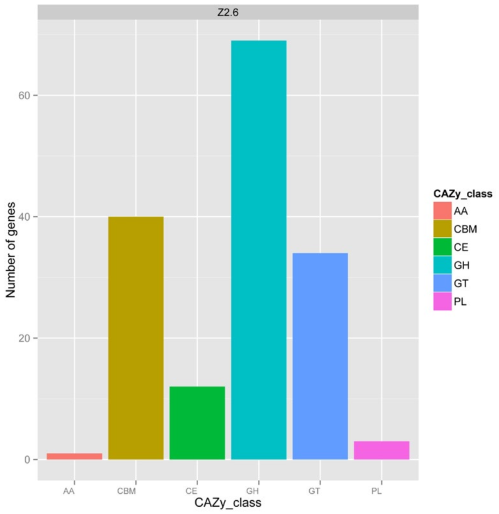

Figure S5. Functional classification map based on the CAZy annotation. Statistical map with the number of genes belonging to the six classification notes based on the CAZy database. Above is the sample ID and the horizontal coordinate is the corresponding classification type, namely Auxiliary Activities (AAs), Carbohydrate-Binding Modules (CBMs), carbohydrate esterases (CEs), glycoside hydrolases (GHs), glycosyl transferases (GTs), and polysaccharide lyases (PLs).

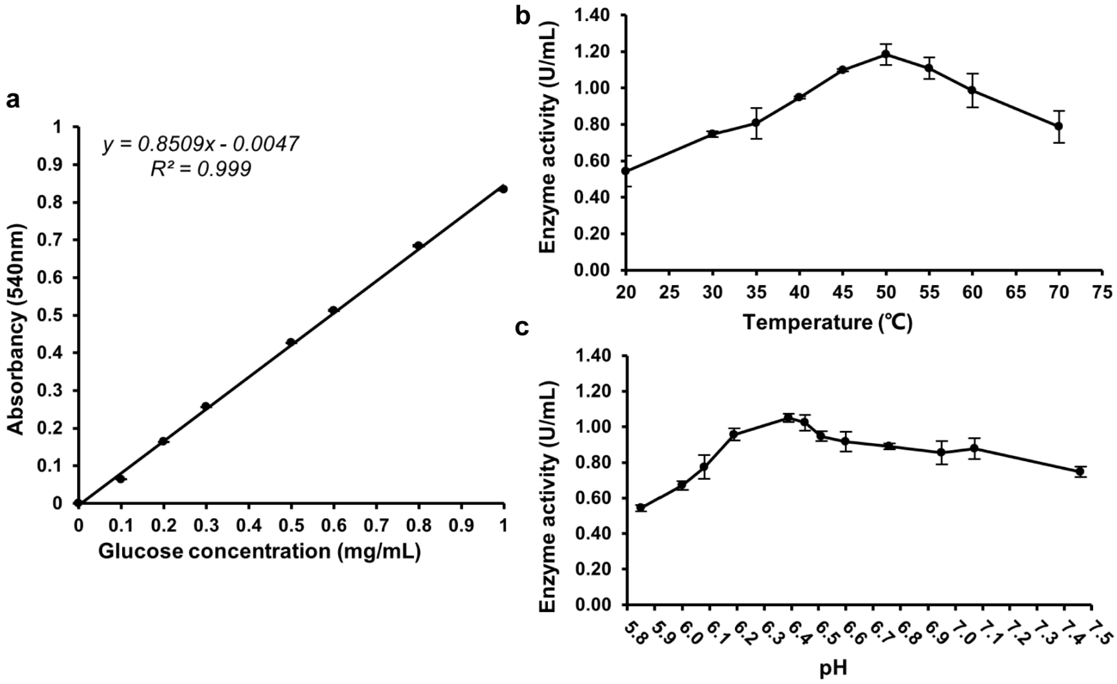

Figure S6. Enzymatic reaction optimum conditions of crude cellulase. A standard curve (a) was regressed by absorbances (OD_{540nm}) versus glucose concentration as the reference in the DNS method, with a determination coefficient (R^2) greater than 0.999. Glucose concentration was diluted from the glucose standard solution (1.0 mg/mL). Optimal enzymatic conditions of **(b)** temperature and **(c)** pH indicate that the optimum temperature and pH for the reaction are approximately 50℃ and 6.49 respectively. Data are all mean ± SEM in triplicates at each point.

Supplementary Tables

Table S1

Premiers used to clone cellulase-related genes in this study.

Locus	Premiers (Forward/Reverse)	Sequence	Tm
V7S33_01155	F	GGATAGAGAGAGGGAGGAAATAATG	58
	R	CGCCAAAATACATATAGACGCTATG	
V7S33_04525	F	CGAAGCGGATGCTTGAAGTG	58
	R	GAAAAGCCGGACAGTCACCT	
V7S33_05150	F	GGACAAAAACGCCAGTAGCC	58
	R	TCATCCGCCACGTAAACCTC	
V7S33_13785	F	TGTCATCTGGCTCCCGTTTC	58
	R	CCAGAATGGTGCCGTCTCTT	
V7S33_13965	F	ATGTTTTATCGTATGAAACGAGTGC	58
	R	TTATTTTTTTGTATAGCGCACCCA	

Table S2

Details of complete randomized design in progressive one-factor-at-a-time screening.

Factor	Gradient or sources								
Carbon Source Categories	Agar	Glucose	Lactose	Maltose	Soluble Starch	CMC-Na	Gelatin	Sodium carbonate	
Nitrogen Source Categories	Yeast extract	$NH_4H_2PO_4$	Beef extract powder	Tryptone	Urea	Casein acid hydrolyzed	KNO_3	NH_4Cl	
CMC-Na	0.05%	0.10%	0.50%	0.75%	1.00%	1.50%	2.00%	2.50%	
Tryptone	0.05%	0.10%	0.30%	0.50%	0.75%	1.00%	1.50%	2.00%	2.50%
Initial pH	3.99	5.02	6.06	6.49	7.05	7,54	8.98	10.02	
Salinity	0.00%	0.30%	0.90%	1.50%	1.95%	2.50%	3.00%	5.00%	
Temperature	20℃	30℃	40℃	50℃	60℃				
Incubation time	From time point 0 h to 120 h at 12 hrs intervals								
Inoculum size	1.0%	2.0%	3.0%	4.0%	5.0%	6.0%			
Bottling size	30 mL	50 mL	80 mL	100 mL	120 mL	150 mL			

Table S3

Parameters and their two levels in PB design. Dummy means dummy variables, set to meet the experimental requirements.

Factor	units	Variables	Levels

			-1	1
CMC-Na	%	A	0.5	1
Tryptone	%	B	0.5	1
Initial pH	-	C	40	45
Temperature	℃	D	40	45
Salinity	%	E	0	1.5
Inoculum size	%	G	2	4
Bottling size	%	H	50	80
Incubation time	h	F	48	72
dummy1*		I		
dummy2*		J		
dummy3*		K		

Table S4

Variables and their levels employed in BBD.

Factor	Units	Variable	Levels		
			-1	0	1
CMC-Na (X_1)	%	A	0.75	1	1.25
Salinity (X_2)	%	E	0.40	0.65	0.90
Tryptone (X_3)	%	B	0.75	1	1.25

Table S5

Spot inoculation with replicas assay for detecting transparent zone. Responses were calculated by the average of triplicate with standard deviation.

Strain	Time [a]	H/C [b]			Mean (SD)
		R1	R2	R3	
Z2.6	1	2.10	2.53	2.74	2.46 ± 0.325
	2	3.21	3.83	3.40	3.48 ± 0.317
	3	3.99	4.78	4.75	4.50 ± 0.450
	4	5.10	5.64	3.99	4.91 ± 0.842
	5	6.14	5.67	4.55	5.45 ± 0.820

[a] Time was converted to unit as day;

[b] The ratio H/C means hydrolytic ring diameter (H) versus colony diameter (D), in which each plate was spotted by 3 single colonies as triplicate (R1–3) with the remaining one as control.

Table S6

Summary of growth curve by nonlinear least square (NLS) in R.

Parameters	Estimate	Std. Error [a]	t-value	Pr(>|t|) [e]
K_alpha [b]	0.123315	0.004597	26.825	4.43E-12 ****
r.scale [c]	0.238242	0.039509	6.03	5.94E-05 ****
N0.alpha [d]	0.017126	0.004713	3.634	0.00343 ***
Residual standard error			0.0101	
Number of iterations to convergence			0	

Achieved convergence tolerance	3.11E-06
Formula	y ~ K * N0 * exp(r * x)/(K + N0 *(exp(r * x) - 1))
Theoretical curve	$Y_{OD_{600nm}} = 1.2468 \times \frac{0.1429 \times e^{0.3261t}}{1.2468 + 0.1429 \times (e^{0.3261t} - 1)}$

[a] Std. Error means standard error;

[b] K alpha is the carrying capacity estimated by original data;

[c] r scale is the instantaneous growth rate.

[d] N0 alpha is the initial population scale by prediction

[e] Significance codes: 0.0001 '****', 0.001 '***', 0.01 '**', 0.05 '*'

Table S7

Annotated genes encoding cellulose-degradation-related enzymes of *Bacillus velezensis* Z2.6 by the CAZy database. "EC#" means the EC recording numbers.

Classification	CAZy	Count	Predicted function	EC#
Cellulase-related	GH51	2	endo-1,4-β-glucanase	3.2.1.4
	GH13_31	3	α-glucosidase	3.2.1.20
	GH1	7	β-glucosidase	3.2.1.21
	GH3	2	β-glucosidase	3.2.1.21
	GH4	1	α-galactosidase	3.2.1.22
	GH32	1	endo-levanase	3.2.1.65
	GH16_21	1	β-1,3(4)-glucanase	3.2.1.73
	GH1	1	6-phospho-β-galactosidase	3.2.1.85
	GH1	2	6- phospho-β-glucosidase	3.2.1.86
	GH4	1	6-phospho-β-glucosidase	3.2.1.86
	GH4	1	6-phospho-α-glucosidase	3.2.1.122
Hemicellulase-related	GH11	1	endo-beta-xylosidase	3.2.1.8
	GH30_8	1	endo-beta-xylosidase	3.2.1.8
	GH43_11	1	1,4-β-xylosidase	3.2.1.37
	GH51_1	2	α-N-arabinofuranosidase	3.2.1.55
	GH43_16	1	α-N-arabinofuranosidase	3.2.1.55
	GH26	1	endo-1,4-β-mannosidase	3.2.1.78
	GH43	2	Arabinan endo-1,5-α-L-arabinosidase	3.2.1.99

Table S8

ANOVA and model evaluation for Plackett–Burman design. Significance codes are 0.05 '*', 0.01 '**', and 0.001 '***', where statistical signification is at the 95% confidence level ($p < 0.05$).

Source	Sum of Squares	DF	Mean of Square	F value	*p*-value Prob > F	
**Model**	4.07	3	1.36	16.99	0.0008***	significant
A: CMC-Na	2.3	1	2.3	28.77	0.0007***	
B: Tryptone	0.7599	1	0.7599	9.52	0.0150*	
E: Salinity	1.01	1	1.01	12.67	0.0074**	
**Residual**	0.6386	8	0.0798			

**Cor total**	4.71	11

Table S9

Responses according to the Box–Behnken method with the summary of ANOVA and model fitness. Significance codes are 0.05 "*" and 0.01 "**"

Source	Sum of Squares	DF	Mean of Square	F value	*p*-value Prob > F [a]	
Model	2.41	9	0.2673	9.14	0.004**	significant
X_1 – CMC-	0.1881	1	0.1881	6.43	0.0389*	
X_2–Salinity	0.1199	1	0.1199	4.1	0.0825	
X_3–Tryptone	0.1503	1	0.1503	5.14	0.0577	
X_1X_2	0.1799	1	0.1799	6.15	0.0422*	
X_1X_3	0.0217	1	0.0217	0.7429	0.4173	
X_2X_3	0.1772	1	0.1772	6.06	0.0433*	
X_1^2	0.703	1	0.703	24.05	0.0017**	
X_2^2	0.6929	1	0.6929	23.7	0.0018**	
X_3^2	0.0464	1	0.0464	1.59	0.248	
Residual	0.2047	7	0.0292			
Lack of Fit	0.1664	3	0.0555	5.8	0.0612	not significant
Pure error	0.0382	4	0.0096			
Cor total	2.61	16				
